# COVID-19 and All-Cause Mortality by Race, Ethnicity, and Age Across Five Periods of the Pandemic in the United States

**DOI:** 10.1007/s11113-023-09817-8

**Published:** 2023-08-03

**Authors:** Anneliese N. Luck, Irma T. Elo, Samuel H. Preston, Eugenio Paglino, Katherine Hempstead, Andrew C. Stokes

**Affiliations:** 1 Department of Sociology and Population Studies Center, University of Pennsylvania, Philadelphia, USA; 2 Robert Wood Johnson Foundation, Princeton, USA; 3 Department of Global Health, Boston University School of Public Health, Boston, USA

**Keywords:** COVID-19, Mortality and morbidity, Heterogeneity, Race/ethnicity, Age

## Abstract

Racial/ethnic and age disparities in COVID-19 and all-cause mortality during 2020 are well documented, but less is known about their evolution over time. We examine changes in age-specific mortality across five pandemic periods in the United States from March 2020 to December 2022 among four racial/ethnic groups (non-Hispanic White, non-Hispanic Black, Hispanic, and non-Hispanic Asian) for ages 35+. We fit Gompertz models to all-cause and COVID-19 death rates by 5-year age groups and construct age-specific racial/ethnic mortality ratios across an Initial peak (Mar–Aug 2020), Winter peak (Nov 2020–Feb 2021), Delta peak (Aug–Oct 2021), Omicron peak (Nov 2021–Feb 2022), and Endemic period (Mar–Dec 2022). We then compare to all-cause patterns observed in 2019. The steep age gradients in COVID-19 mortality in the Initial and Winter peak shifted during the Delta peak, with substantial increases in mortality at working ages, before gradually returning to an older age pattern in the subsequent periods. We find a disproportionate COVID-19 mortality burden on racial and ethnic minority populations early in the pandemic, which led to an increase in all-cause mortality disparities and a temporary elimination of the Hispanic mortality advantage at certain age groups. Mortality disparities narrowed over time, with racial/ethnic all-cause inequalities during the Endemic period generally returning to pre-pandemic levels. Black and Hispanic populations, however, faced a younger age gradient in all-cause mortality in the Endemic period relative to 2019, with younger Hispanic and Black adults in a slightly disadvantageous position and older Black adults in a slightly advantageous position, relative to before the pandemic.

## Introduction

The disproportionate impact of the COVID-19 pandemic on populations of color has garnered significant attention since the onset of the pandemic. A large body of literature has documented stark disparities in infection, hospitalization, and mortality rates across racial/ethnic groups during the first year of the pandemic in the United States (Aburto et al., 2022; Alsan et al., 2021; Andrasfay & Goldman, 2021; Bassett et al., 2020; Luck et al., 2022; Shiels et al., 2021; Truman, 2022; Yuan et al., 2023). Between 2019 and 2020, the decline in life expectancy for Hispanic (4.0 years) and non-Hispanic Black, hereafter Black, populations (3.3 years) was more than double that observed for the non-Hispanic White, hereafter White, population (1.2 years), while non-Hispanic Asian, hereafter Asian, populations lost 2.0 years (Arias & Xu, 2022). Further, studies also show significant differences in the age pattern of the pandemic’s mortality burden across racial/ethnic groups, with particularly large increases observed among younger Black and Hispanic adults (below age 65) relative to their White peers (Bassett et al., 2020; Elo et al., 2022; Luck et al., 2022; Wrigley-Field et al., 2020). The unequal mortality burden of the pandemic in 2020 upended recent progress made over the last decade in reducing the Black-White life expectancy gap and nearly eliminated the long-standing Hispanic life expectancy advantage, termed the Hispanic Mortality Paradox (Andrasfay & Goldman, 2021; Garcia & Sáenz, 2023; Woolf et al., 2021).

While the disproportionate impact of the pandemic on populations of color in 2020 is well documented, research has only recently begun to consider how these inequalities have changed over time. Emerging work has generally focused on changes between 2020 and 2021, with evidence suggesting a narrowing of racial/ethnic gaps in 2021, despite continued decreases in life expectancy among all racial/ethnic groups (Ahmad et al., 2022; Andrasfay & Goldman, 2022; Lundberg et al., 2023). At the same time, prior work has also documented sharp variation in COVID-19 mortality over time, with several mortality peaks and valleys characterizing the pandemic, driven by new variants, trends in vaccination uptake, and the intensity of prevention measures (del Rio & Malani, 2022; Truman, 2022). This literature suggests disaggregating across age groups and over disparate pandemic periods may provide crucial insight into how the pandemic’s uneven effects were distributed across racial/ethnic populations (Aschmann et al., 2022; Elo et al., 2022).

One recent study documented changes in the age pattern of COVID-19 mortality by race and ethnicity over time, finding that death rates increased for younger and declined for older adults across Black, Hispanic, and White subgroups between July–October 2021 relative to earlier periods (Elo et al., 2022). We build on this work in a few crucial ways. First, we include the Asian population, a group generally understudied in COVID-19 literature. Second, we extend the analysis to all-cause mortality, given the growing body of evidence that suggests racial/ethnic variation in non-COVID-19 mortality increased during the pandemic (Aburto et al., 2022; Ahmad & Anderson, 2021; Luck et al., 2022; Woolf et al., 2020). Finally, we analyze more recent pandemic periods, including the Omicron peak, which has been characterized by high infection rates and waning immunity from prior vaccination or infection (Ferdinands et al., 2022), as well as the period after Omicron, when COVID-19 infections and mortality declined. This most recent period offers an introduction to the endemic phase of the pandemic, providing valuable insight into the potential future of COVID-19 racial and ethnic inequality in the United States.

## Background

### Historical Trends in Racial and Ethnic Mortality Inequality

Scholars have long documented substantial variation in mortality and longevity by race and ethnicity in the United States (Hahn & Eberhardt, 1995; Harper et al., 2007; Johnson et al., 2022; Murray et al., 2006; National Academies of Sciences et al., 2017). In 1990, for example, White men could be expected to live nearly 8 years longer than Black men but 9 years shorter than Asian men, while White women could be expected to live 6 years longer than Black women and 6 years shorter than Asian women (Hahn & Eberhardt, 1995). Life expectancy among Hispanic men and women has also historically exceeded that of their non-Hispanic Black and White counterparts. For example, the life expectancy at birth in 2019 of Hispanic men exceeded that of White men by 2.8 years and by 3.1 years for women, while the respective differences between Black and Hispanic men and women were 7.8 years and 6.3 years (Johnson et al., 2022). Despite improvements over time, substantial racial and ethnic variation in mortality persists, with as much as a 10.8-year difference in life expectancy between Asian and Black Americans in 2019 (Arias et al., 2021).

Black Americans, in particular, face a large and persistent mortality disadvantage relative to their peers (Cullen et al., 2012; Cunningham, 2017; Harper et al., 2014). Although the Black-White mortality gap has gradually narrowed over time, Black individuals continue to experience higher mortality rates than their White counterparts across most age groups and causes of death (Currie & Schwandt, 2016; Harper et al., 2007, 2014; Macinko & Elo, 2009; Schwandt et al., 2021). The largest Black–White gaps are in premature mortality, or mortality occurring before the age of 65, in part due to particularly high rates of infant mortality and violence-related deaths at younger ages (Caraballo et al., 2023; Cunningham, 2017; Schwandt et al., 2021). At the very oldest ages, research has found that Black individuals begin to face lower mortality risk than their White peers—a Black–White mortality crossover possibly driven by data quality issues or mortality selection at younger ages (Arias, 2019; Preston et al., 1996). The distinct disadvantage of Black Americans when it comes to health and mortality in the United States has generated a large body of literature concerned with the human costs of structural racism in the United States (Bailey et al., 2017; Gee & Ford, 2011).

Conversely, Hispanic individuals in the United States have experienced a long-standing mortality advantage, with lower all-cause mortality rates than the White population. Because of their lower socioeconomic standing, the mortality advantage of Hispanic over White individuals has been termed the “Hispanic Mortality Paradox” (Franzini et al., 2001; Ruiz et al., 2013). This mortality advantage is particularly pronounced among infants, middle-aged and older adults (Ruiz et al., 2013), as well as among foreign-born Hispanic immigrants (Hummer et al., 1999; Lariscy et al., 2014). One of the main drivers of the Hispanic-White mortality gap are differences in smoking behavior, with lower mortality from smoking related causes of death, such as heart disease, several forms of cancer, and respiratory diseases, among Hispanic populations (Fenelon, 2013). However, the Hispanic Mortality Paradox does not extend uniformly across all causes of death or health outcomes. Hispanic individuals are found to face higher mortality than White individuals from certain causes of death, such as diabetes, liver disease, homicide, as well as stomach, liver, and cervical cancers (McDonald & Paulozzi, 2019). Additionally, Hispanic individuals remain disadvantaged relative to their White peers across many health indicators, living with higher rates of morbidity, disability, stress, and functional limitations, despite their longer life spans (Boen & Hummer, 2019; Crimmins et al., 2007; Hayward et al., 2014; Sheftel & Heiland, 2018).

A smaller body of research has documented a sizable Asian health and mortality advantage in the United States. Historically, Asian Americans have outlived all other racial and ethnic groups, facing lower death rates across nearly all causes of death (Arias et al., 2021; Hahn & Eberhardt, 1995; Murray et al., 2006), a gap which is largest at older ages (Hummer et al., 1999; Lauderdale & Kestenbaum, 2002; Liao et al., 1999). The advantage is also found to be most pronounced among Asia-born immigrants, who have exceptionally high levels of longevity and make a large contribution to the overall Asian overall mortality advantage (Hummer et al., 1999; Mehta et al., 2016). Yet the mortality of Asian Americans remains understudied, particularly relative to their White, Black, and Hispanic peers. The lack of inclusion of Asian populations has largely been due to data quality concerns, particularly around inconsistencies in racial coding between death certificates, in which race is reported by coroners or medical examiners, and population counts, in which race is self-reported (Elo, 1996; Hahn & Eberhardt, 1995; McKenney & Bennett, 1994). However, assessments have indicated that reporting of Asian racial classifications on death certificates have improved substantially over time, with underestimation rates now at around 3 percent overall, or similar to that of the Hispanic population (Arias et al., 2016). Given this improvement in reporting, the National Academies of Sciences, Engineering, and Medicine recently concluded death certificate race data to be of sufficient quality to produce life tables for and to include the Asian populations studies of mortality using vital statistics and census data—an increasingly important task given the rapid growth and the diversity of the Asian population in the United States (Baluran & Patterson, 2021; National Academies of Sciences, Engineering, and Medicine, 2021).

### Racial and Ethnic Variation in Mortality During the COVID-19 Pandemic

Although the United States has a long history of racial and ethnic disparities in mortality, recent research has documented the arrival of unprecedented levels of inequality during the COVID-19 pandemic. Much of this work has focused on the first year of the pandemic, when striking mortality disparities appear to have disrupted recent racial and ethnic trends in longevity. Relative to White life expectancy, which dropped by 1.4 years in 2020, life expectancy among Hispanic, Black, and Asian individuals decreased by 4.0, 3.3, and 2.0 years, respectively (Arias & Xu, 2022). This unequal mortality impact reduced the Asian life expectancy advantage, erased the progress made in the last decade at narrowing the Black-White mortality gap, and nearly eliminated the Hispanic mortality paradox (Andrasfay & Goldman, 2022; Woolf et al., 2021), with a complete disappearance of the paradox recorded in some states (Garcia & Sáenz, 2023).

Mortality directly attributable to COVID-19 played an important role in the unequal impact of the pandemic. Throughout 2020, research documented higher COVID-19 death rates among populations of color, particularly Black and Hispanic populations, relative to their White peers (Aburto et al., 2022; Alsan et al., 2021; Andrasfay & Goldman, 2021; Bassett et al., 2020; Luck et al., 2022; Shiels et al., 2021; Truman, 2022; Yuan et al., 2023). The burden of COVID-19 in 2020 on individuals of color was also younger than that on White individuals (Aburto et al., 2022; Bassett et al., 2020; Luck et al., 2022). For example, during the first half of 2020, Bassett et al (2020) found age-standardized COVID-19 death rates among Black, Hispanic, and Asian individuals were 3.6, 2.8, and 1.6 times higher than those observed among White individuals, respectively. At working-ages (aged 25–64), these ratios increased between 50 to 100 percent. Additionally, these inequalities appear to have shifted even across the first year of the pandemic (Wong et al., 2021). Truman (2022) showed that, while the burden on Black and Hispanic individuals relative to White individuals remained high across the entire 2020 year, the early Asian COVID-19 disadvantage disappeared. In particular, research documented a distinctly heavy burden of COVID-19 mortality on Hispanic individuals during the first year of pandemic, particulaly working-age Hispanic individuals, who experienced some of the largest losses of life from COVID-19 (Aburto et al., 2022; Luck et al., 2023).

This early impact of COVID-19 on communities of color called attention to the many structural factors that placed these populations in harm’s way at the onset of the pandemic (Abraham et al., 2021; Bailey et al., 2017; Brown et al., 2022; Chen & Krieger, 2021; Egede & Walker, 2020; Garcia et al., 2021; Selden & Berdahl, 2020; Tai et al., 2021). In particular, scholars have pointed to the disproportionate risk of COVID-19 infection faced by populations of color, especially Black and Hispanic individuals (Kabarriti et al., 2020; Mackey et al., 2021; Shortreed et al., 2023; Zelner et al., 2021). Though Asian mortality is less studied, research also documented particularly high Asian COVID-19 hospitalization and case fatality rates early in the pandemic (Wang et al., 2020; Yan et al., 2021). This heavy mortality burden on populations of color has been tied to a variety of structural inequalities across employment (Chen et al., 2021, 2022a, 2022b; Goldman et al., 2021; Matthay et al., 2022; Rodriguez-Diaz et al., 2020; Rogers et al., 2020), housing (Chen & Krieger, 2021; Riley et al., 2021), and healthcare (Bleich & Ard, 2021; Cantor et al., 2022; Kim et al., 2021; Mishra et al., 2021; Wiltz, 2022).

However, the pandemic’s mortality impact in 2020 was not limited to deaths attributable to COVID-19. A large body of work also documented substantial increases during the first year of the pandemic in non-COVID-19 mortality across a variety of causes of death, including heart disease, diabetes, drug overdose, and homicide (Aburto et al., 2022; Ahmad & Anderson, 2021; Faust et al., 2021; Luck et al., 2022, 2023; Shiels et al., 2021; Woolf et al., 2020). While Hispanic individuals saw the highest rates of COVID-19 in 2020, Black individuals saw the largest increases in all-cause mortality, with nearly a third of that increase found to be attributable to causes other than COVID-19, such as heart disease, diabetes, and external causes (Chen et al., 2022a, 2022b; Luck et al., 2022). There are a variety of possible factors driving these indirect deaths, including inconsistencies in cause of death coding, inadequate access to care for chronic conditions due to strained hospital systems, hospital avoidance, or the increased despair and stress associated with the financial strain of the pandemic, which may have disproportionately burdened the already disadvantaged Black population (Anderson et al., 2021; Cantor et al., 2022; Chan & Sneed, 2023; Matthay et al., 2021; Stokes et al., 2021).

Less is known about how these racial and ethnic inequalities have changed since the first year of the pandemic. Data from the Center for Disease Control comparing 2021 to 2020 mortality suggest a growing burden of COVID-19 on the White population, as COVID-19 death rates decreased among Black, Hispanic, and Asian populations in 2021 yet increased among White individuals, by an estimated 35% (Ahmad et al., 2022; Truman, 2022). Although Black and Hispanic COVID-19 mortality remained higher than White mortality in 2021, these shifts appear to have narrowed the COVID-19 mortality disadvantage of Black and Hispanic populations while widening the Asian COVID-19 mortality advantage (Ahmad et al., 2022; Andrasfay & Goldman, 2022). Evidence further suggests that the burden of mortality in the second year of the pandemic shifted younger, with age groups under 80 contributing more to life expectancy losses in 2021 relative to 2020 (Schöley et al., 2022).

A small body of work has examined more granular changes over time, finding similar racial/ethnic and age shifts when comparing the initial wave of the pandemic in 2020 to the more recent waves during 2021, such as during the Delta and Omicron variants. For example, Lundberg et al. (2023) found a narrowing in national Black-White COVID-19 mortality gaps between the initial and Omicron waves, while Elo et al. (2022) found that the substantial increase in COVID-19 death rates among younger adults occurred during the Delta wave (July to October 2021) across all racial/ethnic groups, but to a larger degree among White young adults. By Feburary 2022, Aschmann et al (2022) noted a return to prepandemic levels of all-cause mortality among Black and Asian populations, but particularly persistent inequality between younger indigenous adults relative to their White peers (under age 65).

Our understanding of the factors driving the evolution of age and racial/ethnic patterns in mortality remains limited. However, with the dissemination of COVID-19 vaccinations across 2021, racial/ethnic and age patterns in vaccinations rates are likely to be increasingly linked to changing patterns in mortality (Agaku et al., 2022; Gupta et al., 2021; Saelee, 2022). Recent work has also highlighted the importance of the changing geography of COVID-19. Although higher COVID-19 infection and mortality in 2020 was generally associated with greater population density and more racially diverse areas, emerging evidence indicates a shift towards a rural disadvantage as the pandemic progressed (Lundberg et al., 2023; Paglino et al., 2023). For example, Lundberg et al (2023) finds that a majority of the decrease in Black-White COVID-19 disparities observed between the initial and Omicron waves was explained by increases in White mortality and shifts to nonmetropolitan areas where more White individuals reside. Nonetheless, more research on the evolution of mortality inequalities across the pandemic is needed to clarify the potential role these factors played in shaping the evolving mortality burden of the pandemic.

## Data and Methods

We use mortality data from January 2020 through December 2022 from the National Center of Health Statistics (National Center for Health Statistics, 2022) and monthly population estimates for 2020 to 2022 from the US Census Bureau (U.S. Census Bureau, 2022). We classify COVID-19 deaths based on multiples cause of death files in which the COVID-19 code of U07.1 was mentioned as an underlying or contributing cause of death using the *International Statistical Classification of Diseases and Related Health Problems, Tenth Revision*.

**Fig. 1:**
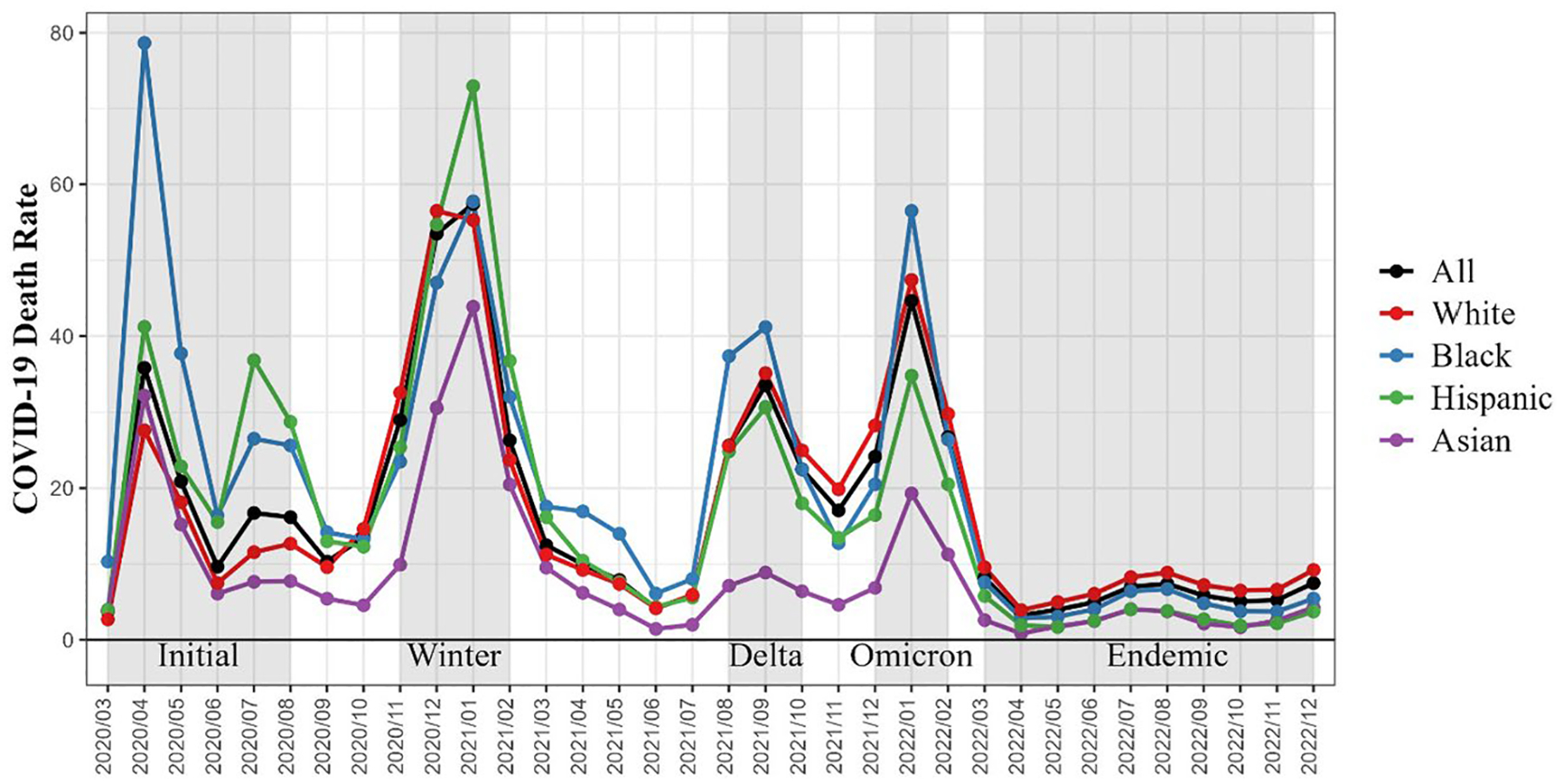
COVID-19 crude death rates by race/ethnicity between 1/2020 and 12/2022. Note: Death rate per 100,000 for population aged 35+. Shaded areas indicate mortality periods thorughout COVID-19 pandemic selected for analyses

Monthly death counts were obtained for the US adult population aged 35+ by 5-year age groups (35–39, … 80–84, 85+), sex, ethnicity, and race. Given that death counts below 10 are suppressed for any given category in the publicly available data, we limited the analysis to ages 35+ to avoid data suppression at the youngest ages, particularly among Asian populations. Peaks in COVID-19 mortality were visually identified by steeply rising monthly COVID-19 crude death rates followed by steep declines and a relatively low death rate. Figure 1 shows COVID-19 death rates by month for the population aged 35+ from January 2020 through December 2022 by race/ethnicity. We identified five pandemic periods for analysis: four mortality peaks and a most recent period of lower mortality. These five periods are the six-month peak from 3/2020 to 8/2020 (Initial), the four-month peak from 11/2020 to 2/2021 (Winter), the three-month peak from 8/2021 to 10/2021 (Delta), the four-month peak from 12/2021 to 2/2022 (Omicron), and the recent ten-month period of lower mortality from 3/2022 to 12/2022 (Endemic). All-cause and COVID-19 mortality data were re-extracted for these five pandemic periods to further mitigate suppression that may be occurring at the monthly level. Analyses were stratified by race/ethnicity for Hispanic, non-Hispanic Black (referred to as Black), non-Hispanic White (referred to as White), and non-Hispanic Asian (referred to as Asian) populations, based on single-race coding across both mortality and population data sources. We calculate annualized all-cause and COVID-19 age- and race/ethnicity-specific death rates by dividing the total number of deaths in each period by the total person-years lived.

To examine the age pattern of mortality by race/ethnicity across pandemic periods, we fit Gompertz mortality curves to age-specific death rates across the periods separately for all racial/ethnic groups. The classic Gompertz mortality curve, which describes adult all-cause mortality rates, assumes that mortality increases linearly with age on a logarithmic scale (Gompertz, 1825; Sasson, 2021). Prior studies have established the applicability of Gompertz curves to COVID-19 mortality, with the age patterns of COVID-19 resembling that of all-cause mortality by increasing exponentially with age (Goldstein & Lee, 2020; Promislow, 2020).

To analyze changes across the pandemic periods, we estimate the following model separately by race/ethnicity. Following established approaches in the literature (Horiuchi & Wilmoth, 1998; Tai & Noymer, 2018), the open-ended age interval (85+) is excluded from the Gompertz models. Let us denote 5-year mortality rates for age group [*x*, *x* + 5) and period *p* with *m*_*x*,*p*_

$$\log\left(m_{x,p}\right)=\alpha +\beta^{A}\cdot (x+2.5)+\underset{p}{\sum }\left(\beta_{p}^{P}+\beta_{p}^{AP}\cdot (x+2.5)\right)\cdot \text{Perio}{\text{d}}_{p}+\varepsilon_{x,p}$$

The model is estimated separately for all-cause mortality and COVID-19 mortality. For both all-cause and COVID-19 mortality estimates, we use the 2019 all-cause mortality as a reference for period intercepts and interactions between age and period with the period referring to the five pandemic periods. This strategy allows us to test how age patterns (slopes) of all-cause mortality and COVID-19 mortality differed from the 2019 age pattern of all-cause mortality during the five periods of the pandemic with reference to a common baseline. Note that the 2019 baseline all-cause mortality is specific to each race/ethnic group. Below is a summary of the parameters of the above equation:

α= the intercept of the Gompertz all −cause mortality curve at baseline in 2019

$$\beta^{A}=\text{ the slope of the Gompertz all }-\text{cause mortality curve at baseline in }2019$$

$$\beta_{p}^{P}=\text{ the change in the intercept for period }p\text{ relative to baseline in }2019$$

$$\beta_{p}^{AP}=\text{ the difference in slope for period }p\text{ relative to baseline in to the all - cause mortality slope at baseline in }2019$$

Additionally, we constructed racial/ethnic death rate ratios by dividing age-specific death rates in each period for Black, Hispanic, and Asian populations by corresponding age-specific death rates in the same period for the White population. To estimate confidence intervals around these death rate ratios, standard errors for each age- and race/ethnicity-specific deaths were derived using the formulas in Chiang (1984). We first drew 1000 samples from the asymptotic distribution of each mortality rate and then used these samples to simulate age-specific racial/ethnic death rate ratios. Finally, we computed empirical percentiles (2.5th and 97.5th) and report these as the lower and upper bounds for each ratio. This approach assumes that death counts for each race/ethnicity, cause of death, and age group follow independent Binomial distributions with probabilities _*n*_*q*_*x*_ (computed separately for each group using standard life-table methods) and where the underlying population size is known.

## Results

### Changing Age Pattern of Mortality by Race/Ethnicity Across the Pandemic Periods

**Fig. 2:**
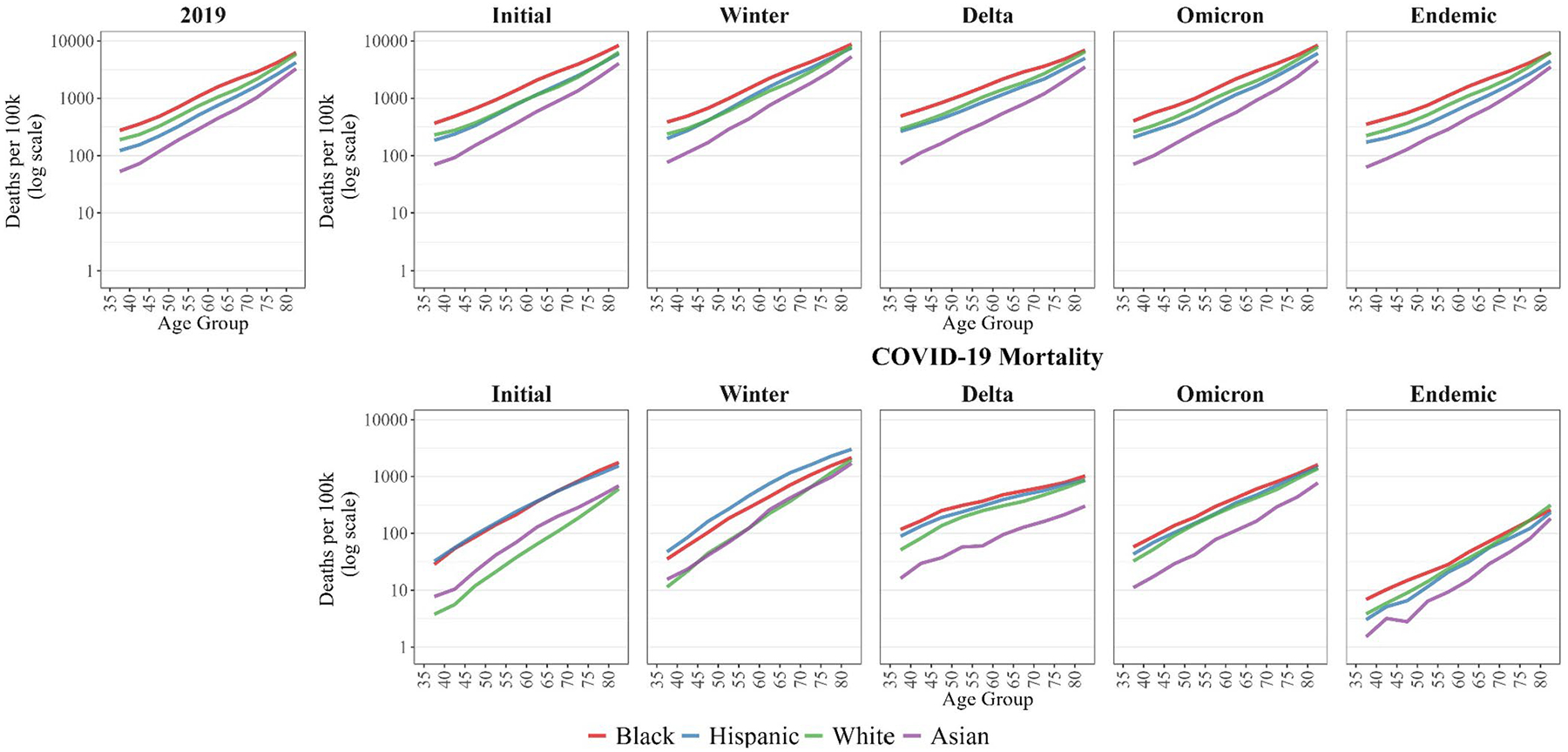
Gompertz curves by race/ethnicity during selected mortality periods. Note: Initial Peak corresponds to 3/2020–8/2020, Winter Peak to 11/2020–2/2021, Delta Peak to 8/2021–10/2021, Omicron Peak to 12/2021–2/2022, and Endemic Stage to 3/2022–12/2022. Curves plotted at mid-point of 5-year age groups for ages 35–84, with open-age interval (85+) excluded from Gompertz models

Age-specific death rates by race/ethnicity are shown in Table 1. Figure 2 presents Gompertz curves by race/ethnicity for the 2019 baseline period for all-cause mortality and each of the five pandemic periods for all-cause and COVID-19 mortality. The corresponding all-cause and COVID-19 slopes for each racial/ethnic group across the pandemic periods are presented in Table 2. Compared to pre-pandemic all-cause mortality, the steeper slopes of COVID-19 mortality in the early periods of the pandemic reflect the fact that the burden of COVID-19 mortality fell especially hard on older ages (Table 2). This was particularly true among the White population, with slopes during the first two peaks nearly 1.5 times steeper than baseline all-cause mortality age patterns in 2019, as compared to 1.3 times steeper for the Black population and between 1.1 and 1.2 times steeper for both Hispanic and Asian populations.

However, during the Delta period, the slopes of COVID-19 mortality flatten noticeably, indicating a dramatic change in the age pattern of mortality relative to both first two peaks (Fig. 2; Table 2). This shift reflects declines in COVID-19 death rates at older ages and continued increases in death rates at younger ages (Table 1), a shift towards a younger age distribution of COVID-19 mortality. However, COVID-19 slopes begin to steepen again during the Omicron peak, where the age pattern of mortality approached that observed at baseline. By the Endemic period, an older age pattern of COVID-19 mortality closer to what is seen early in the pandemic returns, with slopes between 1.1 to 1.3 times steeper than baseline patterns in 2019.

This dramatic shift in the age pattern of mortality during Delta is also notable for all-cause mortality. For the most part, the age pattern of all-cause mortality throughout the pandemic remains largely similar to pre-pandemic baseline patterns, despite the consistently older age distribution of COVID-19 mortality. During Delta, however, all-cause slopes flatten dramatically across all racial/ethnic groups, reflecting a statistically younger age pattern of all-cause mortality in that period among all populations relative to pre-pandemic levels. Subsequently, all-cause patterns return to pre-pandemic levels. Two key exceptions are Black and Hispanic populations in the most recent Endemic period, whose slopes remain statistically lower than baseline, reflecting the persistence of a younger age pattern in all-cause mortality for these populations, relative to before the pandemic. Apart from the Delta period, COVID-19 slopes are higher than all-cause slopes for all groups and periods.

### Changing Age-Specific Racial/Ethnic Mortality Disparities Across the Pandemic

While the slopes of the Gompertz curves in Fig. 2 illustrate variation in the age pattern of mortality across the pandemic peaks, the racial/ethnic ordering of the curves also provides insights into how the racial/ethnic variation in the level of mortality evolved across the pandemic. Notably, Black and Hispanic populations consistently experienced the highest COVID-19 death rates (Table 1; Fig. 2). In fact, Hispanic individuals below the age of 50 during the Initial peak and across all age groups during the Winter peak experienced the highest COVID-19 death rates, even higher than their Black peers. Importantly, Black individuals still experienced the highest levels of all-cause mortality during these periods and across the entire pandemic. Asian COVID-19 death rates were higher than White death rates during the Initial peak and similar to White death rates during the Winter peak, yet they still experienced the lowest levels of all-cause mortality throughout. As evident from Fig. 2 and Table 1, White COVID-19 mortality remained lower than that of other populations early in the pandemic but increased over time.

**Fig. 3:**
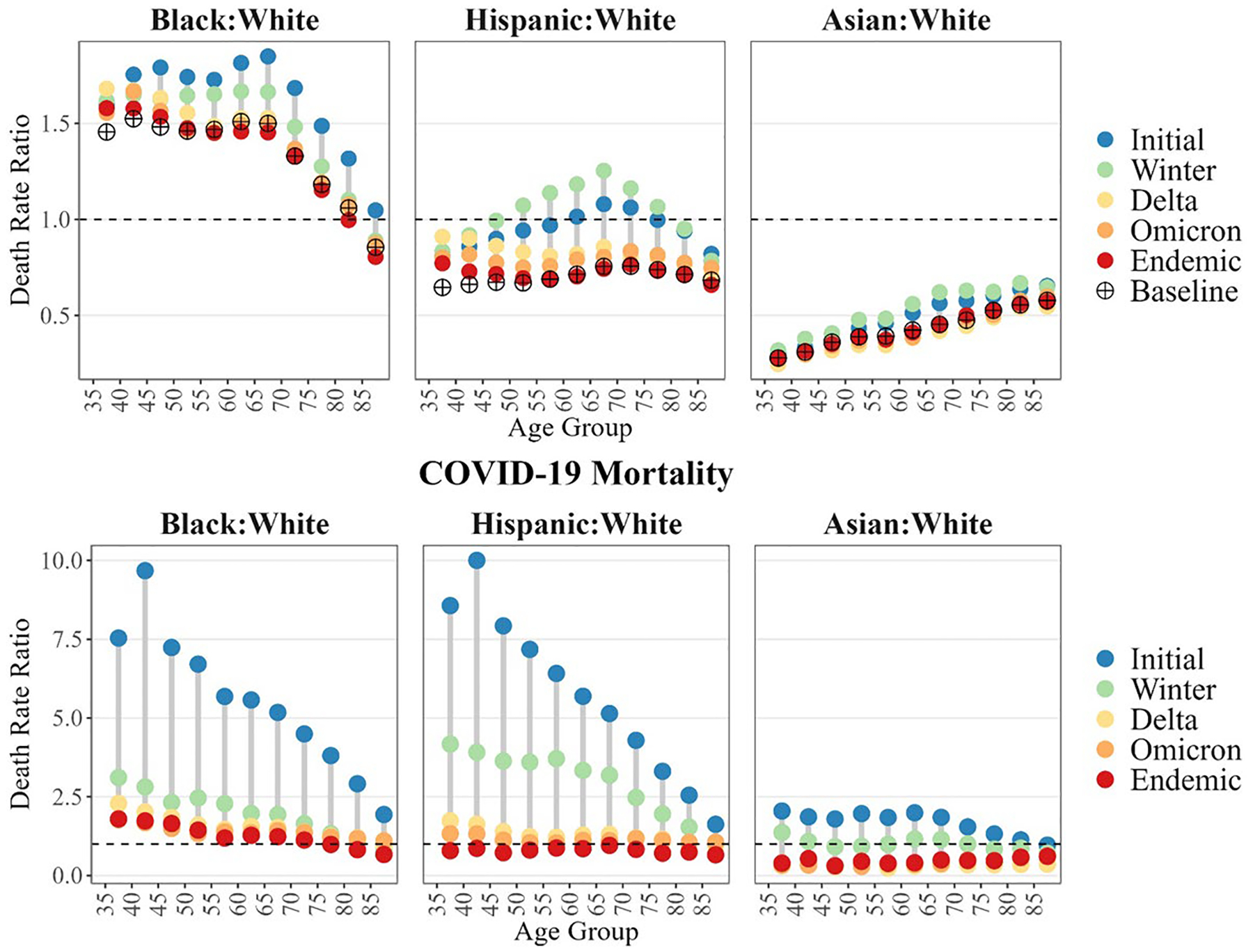
Ratios of race/ethnicity- and age-specific All-Cause and COVID-19 death rates during selected mortality periods. Baseline corresponds to mortality ratios in 2019, Initial Peak to 3/2020–8/2020, Winter Peak to 11/2020–2/2021, Delta Peak to 8/2021–10/2021, Omicron Peak to 12/2021–2/2022, and Endemic stage to 3/2022–12/2022. Ratios plotted at mid-point of 5-year age groups for ages 35–84 and at age 87.5 for open-ended age interval 85+

To examine how age-specific racial/ethnic disparities in COVID-19 and all-cause mortality evolved over time, we present age-specific ratios of all-cause and COVID-19 mortality among Black, Hispanic, and Asian populations to that of the White population in Fig. 3 and Table 3. The full set of racial/ethnic ratios with their corresponding 95% confidence intervals can be found in Appendix Table S1.

The disproportionate mortality burden of the Initial peak of the pandemic on Black, Hispanic and Asian populations relative to the White population across all age groups is clearly seen in Fig. 3. The most pronounced impact was placed on younger Black and Hispanic adults. For example, Black and Hispanic individuals aged 40–44 faced COVID-19 death rates around 10 times higher than White individuals of the same age. Across the Initial and Winter peaks, Black and Hispanic populations continued to face higher COVID-19 mortality than the White population. The impact of the Initial and Winter peaks is also evident in all-cause mortality, with the ratios of Black, Hispanic and Asian age-specific death rates to White rates being significantly higher than in 2019, driving an exacerbation of Black–White inequalities in all-cause mortality and a reduction of the Asian and Hispanic all-cause mortality advantage. In fact, the heavy impact of COVID-19 mortality on Hispanic individuals is reflected in the temporary elimination of any Hispanic all-cause mortality advantage (i.e., the Hispanic Mortality Paradox) between ages 60–79 in the Initial peak and ages 50–79 in the Winter peak. The disproportionate burden of COVID-19 on Black and Hispanic adults, particularly at younger ages, persisted through Omicron, with a COVID-19 disadvantage relative to the White populations across all ages up to this peak.

These striking mortality disparities in the Initial and Winter peaks declined as the pandemic unfolded. Black–White COVID-19 age-specific mortality ratios, which ranged from 1.9 to 9.7 in the Initial peak declined to 1.1 to 1.8 during the Omicron peak. Hispanic-White ratios declined over this same period from 1.6–10.0 to 1.1–1.4 and Asian-White ratios from 1.0–2.1 to 0.3–0.6 (Table 3). The decline was fastest among the Asian population, who gained a COVID-19 advantage across some age groups by the Winter peak and across all age groups by Delta. Large increases in COVID-19 mortality among the White population were partially responsible for the narrowing of these disparities. Comparing the Initial to the Omicron peak, declines in Black and Hispanic mortality from COVID-19 combined with increases in White COVID-19 mortality resulted in the convergence of racial/ethnic gaps at older ages. At younger ages, on the other hand, all racial/ethnic groups experienced increases in COVID-19 mortality, but these increases were relatively larger among the White population than among Black and Hispanic populations (Table 1).

By the Endemic period, all-cause mortality ratios between Asian and White populations had returned to 2019 baseline levels across nearly all age groups, signaling a return to a pre-pandemic Asian mortality advantage (Table 3). An Asian advantage was also obvious for COVID-19 mortality. The Hispanic-White COVID-19 mortality ratios in the Endemic period dropped below one across all age groups, indicating a return to Hispanic mortality advantage, although this COVID-19 advantage remained significantly smaller than their pre-pandemic all-cause mortality advantage for most age groups under age 75. While a similar return of the Hispanic all-cause mortality advantage (i.e., the Hispanic Mortality Paradox) took place among older Hispanic adults (ages 55+), the advantage did not return to the same extent for younger adults, remaining statistically smaller at ages 35–54 than in 2019.

During much of the pandemic, the Black population experienced an exacerbation of their existing mortality disadvantage. By the Endemic period, all-cause and COVID-19 Black-White ratios for those aged 35–54 remained higher than baseline ratios, with significance for all-cause mortality in the age group 35–39 and 45–49. Conversely, Black-White COVID-19 mortality ratios for all ages above 55 and all-cause mortality ratios for most age groups above 60 were lower than in 2019. Notably, these ratios remained above one for all Black age groups, except at the very oldest ages (ages 85+ for all-cause mortality and ages 75+ for COVID-19 mortality). These findings suggests age variation in the return to pre-pandemic levels of all-cause inequality among Black individuals, with slightly larger disadvantages observed among younger Black adults, slightly smaller disadvantages among older Black adults, and a slightly larger advantage at the oldest age group compared to 2019.

## Discussion

This paper examines changing age patterns of COVID-19 and all-cause mortality across racial/ethnic groups during the pandemic and includes the most recent period of decreased mortality as a particularly salient period for anticipating the United States’ endemic COVID-19 future. Relative to 2019 all-cause mortality age patterns, we find evidence of a disproportionate burden of COVID-19 mortality on older adults, with a notable shift towards a younger age pattern in mortality during the Delta peak, and a subsequent gradual return to the early pandemic older age pattern of mortality during Omicron and the Endemic period. We also find evidence of a heavy burden of the early pandemic on Black, Hispanic, and Asian populations, particularly among younger adults. COVID-19 mortality was particularly high among Black and Hispanic populations during the Initial and Winter peaks, resulting in the temporary elimination of the Hispanic Mortality Paradox in all-cause mortality between ages 50 and 79 and the elimination of most of the gain made by the Black population relative to the White population in recent years. The subsequent narrowing of the Black-White and Hispanic-White disparities were the result of the relatively larger increases in White than Black or Hispanic mortality at younger ages, whereas at older ages the reductions were due to relatively larger declines in Black and Hispanic mortality.

Finally, we uncover evidence of a return to the 2019 baseline age pattern of racial/ethnic disparities in mortality during in the most recent Endemic period, with similar pre-pandemic levels of Asian and Hispanic advantage and Black disadvantage reappearing in all-cause mortality. However, we document notable age variation in this return. Young Hispanic and Black individuals (ages 35–54) appear to lag behind their older peers, with young Hispanic adults experiencing a statistically smaller advantage and young Black adults experiencing a larger disadvantage relative to the White population than was the case before the pandemic. This is reflected in the Gompertz models, with Black and Hispanic slopes in the most recent Endemic period remaining statistically lower than baseline age patterns, reflecting the persistence of a younger age pattern in all-cause mortality for these populations relative to before the pandemic. In contrast, the relative Black-White disadvantage at older ages (ages 55–84) appears smaller and the Black-White mortality crossover advantage at the oldest age group (85+) appears larger than in 2019.

These documented shifts in both the age and racial/ethnic patterns of COVID-19 mortality may reflect the combined effects of changes in the nature of the virus, in disease exposure, and in behavior, with most important behavior likely being vaccination (Gupta et al., 2021). Work from the Center for Disease Control suggests that older adults (65+) were much more likely to have been vaccinated by April 2021, between the Winter and the Delta peaks, than younger adults (Kriss et al., 2022), helping to explain the sharp decline in death rates at older ages relative to younger ages between the Winter and the Delta peaks. Subsequent to the Delta period, the age differences in vaccine coverage narrowed considerably. This narrowing is consistent with the re-establishment of a steeper age-pattern of mortality by the Omicron phase but may not account for the increase in mortality at ages 70+ in all racial/ethnic groups. Another factor that could have contributed to the re-establishment of a steeper age pattern during Omicron is waning immunity from both prior infections and vaccinations (Ferdinands et al., 2022). While vaccine boosters became widely available during this period, their uptake was limited compared to the initial vaccine series (Agaku et al., 2022). Additionally, the reduced severity of the Omicron variant and improved treatment may have led mortality to be more heavily concentrated among less healthy older individuals.

Differences in vaccination rates by race/ethnicity are also consistent with the changing patterns in mortality. Asian adults above 30 had the highest vaccine coverage in April 2021, with their advantage over White adults widening by November 2021 (Kriss et al., 2022). Further, although the White population had higher vaccine coverage at every age than the Black or the Hispanic population in April 2021, by November, Hispanic vaccine coverage exceeded that of the White population at all ages and Black coverage exceeded White coverage at ages 30–64 and fell short at ages 65+ by only 1.2% (Kriss et al., 2022). These patterns are consistent with the lowering of Asian mortality to levels below White mortality and the narrowing of disparities between Black and Hispanic populations compared to the White population during the Delta and Omicron peaks. The close correspondence between vaccination rates and trends in mortality by age and race/ethnicity suggests that vaccination has played a major role not just in mortality decline but also in the reduction of racial/ethnic disparities.

Additionally, research has begun to document shifts in the spatial distribution of COVID-19. One recent study examined COVID-19 mortality by race and ethnicity in US metropolitan and non-metropolitan areas over the first two years of the pandemic and found that the national decline in racial/ethnic disparities coincided with shifts in the geography of the pandemic to non-metropolitan areas where increases in White mortality was especially pronounced (Lundberg et al., 2023). The spread of the pandemic to Western and Southwestern states between the Initial and Winter peak is also consistent with the increase in mortality among the Hispanic population, and its subsequent spread to the South and nonmetropolitan areas during the Delta peak is also reflected in the increase in White COVID-19 mortality (Paglino et al., 2023). These shifts are at least in part associated with geographic variation in vaccination rates (Paglino et al., 2023; Saelee, 2022), highlighting the increasingly important role that politics and, in particular, partisan variation in vaccine hesitancy may play in the evolution of COVID-19 mortality patterns (Kaashoek et al., 2022; Sáenz & Garcia, 2022). Although some evidence suggests that these geographic shifts cannot fully explain the changing racial/ethnic disparities (Lawton et al., 2021), the changing geography of COVID-19 and its implications for racial/ethnic disparities in COVID-19 mortality is a promising avenue for future research.

Our study also confirms the heavy burden of mortality imposed on populations of color. Early in the pandemic, the disproportionate burden of COVID-19 mortality on Black, Hispanic, and Asian populations relative to their White peers exacerbated the existing Black all-cause mortality disadvantage and reduced the Hispanic and Asian advantage, with an erasure of the Hispanic Mortality Paradox at ages 60–79 during the Initial peak and ages 50–79 during the Winter peak. Despite considerable narrowing across the pandemic, the Black and Hispanic COVID-19 mortality disadvantage remained persistent across the pandemic. By the Endemic period, the return to pre-pandemic levels of all-cause mortality inequality did not occur to the same extent for younger Hispanic and Black adults, and the age pattern of Black and Hispanic all-cause mortality remained younger than that which was observed before the pandemic.

This burden of the pandemic on populations of color, and the enduring burden on younger Black and Hispanic populations, reflects the continued impact of everyday structural inequalities on the lives of marginalized populations (Macias Gil et al., 2020; McClure et al., 2020), reinforcing structural racism as a fundamental cause of health inequity (Bailey et al., 2017; Phelan & Link, 2015). Communities of color, particularly Black and Hispanic communities, absorbed the overwhelming share of early exposure to the virus (Kabarriti et al., 2020; Mackey et al., 2021; Shortreed et al., 2023; Zelner et al., 2021), likely tied to employment in occupations deemed “essential” (Goldman et al., 2021; Rodriguez-Diaz et al., 2020; Rogers et al., 2020) or residence in multigenerational or overcrowded housing (Chen & Krieger, 2021; Riley et al., 2021). Unequal exposure to the virus may have been further exacerbated by inequalities in healthcare. Studies show Black and Hispanic populations, in particular, more frequently live with the comorbidities associated with severe infection (Bleich & Ard, 2021; Cantor et al., 2022; Kim et al., 2021), lack the financial resources or health insurance needed to access to quality medical care (Macias Gil et al., 2020; Mishra et al., 2021) and receive less adequate COVID-19 treatment when able to obtain care (Wiltz, 2022). The early Asian COVID-19 disadvantage in the Initial and Winter peaks, on the other hand, may be tied to high Asian COVID-19 hospitalization and case fatality rates, possibly due in part to the rise of anti-Asian racism which may have driven hospital avoidance and greater disease severity at care presentation (Wang et al., 2020; Yan et al., 2021).

This analysis has several limitations. First, our analyses relied on provisional mortality data for 2022, which may be subject to incomplete reporting in recent months (Ahmad et al., 2021). To mitigate these concerns, we used a five-month lag between data extraction and the end of the Endemic period included in the analysis. Second, we did not impose formal statistical criteria for identifying COVID-19 mortality peaks and it is possible that use of different definitions of peak periods would have yielded slightly different results. However, we find that the identified four peak periods account for nearly 84% of all COVID-19 deaths and nearly 73% of all all-cause deaths that occurred between January 2020 and December 2022. Third, we have not provided separate estimates by sex. Although the level of COVID-19 and all-cause mortality vary by sex, an earlier study demonstrated a similar evolution in the age pattern of mortality by sex among Black, Hispanic and White individuals through the Delta peak (Elo et al., 2022). Finally, we did not include the American Indian and Alaskan Native population due to data quality issues associated with matching death records and census data (National Academies of Sciences, Engineering, and Medicine, 2021), despite evidence that this group has been heavily affected by the pandemic (Goldman & Andrasfay, 2022; Truman, 2022). Additional research is needed to elucidate patterns of COVID-19 mortality by age among this population.

Nonetheless, this study demonstrates that major age and racial/ethnic changes in COVID-19 mortality patterns occurred as the pandemic evolved, with the patterns observed here highlighting the growing importance of vaccination and the enduring impact of structural racism on exposure to infection, access to health care, and the financial ability to take protective measures, particularly for young Black and Hispanic adults. By documenting these patterns over time, especially into the most recent period of decreased mortality, this study provides crucial insight into the United States’ endemic COVID-19 future.

## Supplementary Material

Supplementary-Appendix

## Figures and Tables

**Table 1:** Age-specific all-cause and COVID-19 death rates (per 100,000) by race/ethnicity during selected mortality periods

	All-cause						COVID-19				
	Baseline	Initial	Winter	Delta	Omicron	Endemic	Initial	Winter	Delta	Omicron	Endemic
	(2019)	(3/20–8/20)	(11/20–2/21)	(8/21–10/21)	(12/21–2/22)	(3/22–12/22)	(3/20–8/20)	(11/20–2/21)	(8/21–10/21)	(12/21–2/22)	(3/22–12/22)
**White**											
35–39	189.5	229.9	238.1	291.0	259.2	222.8	3.8	11.4	51.2	32.4	3.8
40–44	233.6	277.3	298.3	380.6	335.7	280.0	5.6	21.7	82.5	52.8	5.9
45–49	325.3	372.4	414.6	514.0	462.9	365.0	12.0	44.8	136.3	91.6	9.0
50–54	485.6	539.6	602.5	729.2	677.2	514.4	21.1	74.4	193.1	144.2	14.2
55–59	733.9	801.9	906.2	1049.4	1018.8	768.6	37.7	124.2	250.8	214.7	23.7
60–64	1052.5	1146.2	1348.2	1431.0	1463.8	1115.0	64.3	225.6	306.1	309.4	36.6
65–69	1453.1	1573.4	1902.3	1895.1	2024.1	1540.0	107.3	366.3	367.3	424.7	58.3
70–74	2179.2	2339.5	2914.0	2695.3	2960.4	2259.0	183.8	649.1	475.7	595.1	98.2
75–79	3514.3	3804.1	4782.6	4140.1	4750.0	3668.3	329.3	1165.0	629.2	919.3	172.1
80–84	5924.7	6381.9	8010.7	6542.7	7877.8	6293.9	606.2	1964.6	857.4	1380.9	314.2
85+	14,072.0	15,262.7	18,767.0	14,544.1	18,597.2	15,630.4	1547.6	4406.0	1380.5	2646.8	853.6
**Black**											
35–39	275.8	367.0	385.3	489.1	403.0	351.8	28.4	35.4	117.2	57.7	6.9
40–44	356.0	486.4	491.4	636.6	559.4	441.6	54.2	61.0	166.5	88.6	10.3
45–49	481.9	667.1	674.3	838.8	724.8	559.9	86.8	104.1	251.1	137.2	14.8
50–54	708.9	940.0	991.2	1133.4	992.0	758.6	141.5	183.2	310.1	192.0	20.4
55–59	1077.7	1384.8	1496.6	1560.9	1476.9	1115.0	214.3	284.0	367.6	297.2	28.3
60–64	1588.4	2079.9	2247.3	2185.5	2184.7	1625.0	358.4	444.6	478.0	419.8	46.9
65–69	2179.4	2908.8	3165.1	2896.3	3020.3	2237.6	555.6	708.8	560.9	604.3	72.0
70–74	2898.5	3940.3	4317.7	3632.0	4048.4	3011.3	826.6	1068.2	656.6	812.7	110.8
75–79	4156.5	5657.7	6101.6	4852.4	5666.4	4231.1	1253.8	1546.8	784.7	1117.0	170.3
80–84	6278.6	8406.2	8829.0	6959.3	8489.2	6275.0	1767.2	2145.6	1025.5	1622.3	258.7
85+	12,031.2	15,989.1	16,699.5	12,427.1	16,284.9	12,587.6	3000.0	3572.7	1347.5	2912.4	573.1
**Hispanic**											
35–39	122.5	185.4	198.6	264.9	208.0	172.0	32.3	47.4	89.0	43.1	3.0
40–44	154.6	238.2	273.9	342.8	274.4	204.1	56.0	84.7	134.4	69.9	5.1
45–49	219.1	334.9	412.1	443.3	358.8	261.5	95.0	162.9	190.0	103.2	6.5
50–54	325.3	508.8	646.4	605.3	508.3	357.1	151.4	267.7	238.7	150.9	11.5
55–59	506.2	777.4	1031.7	851.1	772.6	527.6	241.9	461.0	305.3	222.6	20.8
60–64	751.9	1163.1	1595.5	1174.2	1159.5	784.2	366.0	752.2	393.4	345.2	31.4
65–69	1098.1	1699.1	2385.0	1624.3	1632.2	1143.9	551.8	1166.8	481.2	471.3	55.9
70–74	1649.6	2484.9	3385.6	2174.2	2474.5	1728.8	789.1	1607.6	566.7	699.4	81.9
75–79	2594.1	3794.9	5098.0	3321.3	3873.4	2687.5	1089.8	2280.0	727.0	1017.9	122.5
80–84	4230.2	6004.9	7626.0	5013.8	6107.1	4481.4	1547.2	3023.1	870.2	1455.7	235.4
85+	9627.2	12,533.9	14,726.4	10,284.3	13,924.8	10,311.2	2519.2	4380.5	1325.1	2796.2	564.8
**Asian**											
35–39	52.9	69.6	76.0	72.0	70.5	62.7	7.7	15.6	16.1	11.1	1.5
40–44	72.5	92.5	113.3	113.2	99.8	88.4	10.4	23.5	29.6	17.5	3.2
45–49	117.7	151.3	169.1	163.8	158.9	128.6	21.5	41.2	37.2	29.2	2.8
50–54	188.8	234.8	288.2	252.4	250.1	200.2	41.5	69.3	57.5	42.1	6.4
55–59	288.3	368.4	439.0	362.5	383.2	287.8	69.6	123.7	60.2	77.7	9.3
60–64	446.7	588.7	755.5	548.9	566.2	459.3	128.2	261.8	94.9	112.2	15.0
65–69	659.4	888.2	1182.3	796.6	922.1	691.4	198.5	420.7	128.4	163.7	29.1
70–74	1036.5	1350.7	1836.0	1200.9	1431.2	1133.5	284.0	650.1	162.4	293.7	46.9
75–79	1851.4	2299.2	2985.5	2027.6	2392.8	1932.0	435.8	977.2	214.2	437.7	81.5
80–84	3290.7	4066.1	5362.5	3537.1	4556.8	3538.3	687.1	1706.5	301.8	773.8	183.0
85+	8141.2	9990.5	12,133.7	7949.8	11,155.0	8922.1	1496.0	3125.4	498.5	1580.9	523.6

**Table 2:** Gompertz model slopes and significance tests relative to baseline

	All-cause		COVID-19	
	Beta	*p*-value	Beta	*p*-value
**White**				
Baseline, 2019	0.077	Ref	–	–
Initial, 3/20–8/20	0.074	0.409	0.113	0.000
Winter, 11/20–2/21	0.078	0.546	0.113	0.000
Delta, 8/21–10/21	0.068	0.009	0.058	0.000
Omicron, 11/21–2/22	0.075	0.720	0.081	0.159
Endemic, 3/22–8/22	0.074	0.378	0.097	0.000
**Black**				
Baseline, 2019	0.070	Ref	–	–
Initial, 3/20–8/20	0.070	0.985	0.091	0.000
Winter, 11/20–2/21	0.072	0.456	0.092	0.000
Delta, 8/21–10/21	0.059	0.000	0.045	0.000
Omicron, 11/21–2/22	0.068	0.100	0.073	0.282
Endemic, 3/22–8/22	0.065	0.001	0.081	0.000
**Hispanic**				
Baseline, 2019	0.080	Ref	–	–
Initial, 3/20–8/20	0.078	0.632	0.085	0.100
Winter, 11/20–2/21	0.083	0.266	0.093	0.000
Delta, 8/21–10/21	0.065	0.000	0.049	0.000
Omicron, 11/21–2/22	0.076	0.138	0.077	0.500
Endemic, 3/22–8/22	0.074	0.024	0.096	0.000
**Asian**	0.074			
Baseline, 2019	0.091	Ref	–	–
Initial, 3/20–8/20	0.090	0.840	0.103	0.005
Winter, 11/20–2/21	0.094	0.160	0.107	0.000
Delta, 8/21–10/21	0.084	0.007	0.061	0.000
Omicron, 11/21–2/22	0.091	0.955	0.093	0.591
Endemic, 3/22–8/22	0.088	0.349	0.103	0.004

To estimate Gompertz curve slopes, the log of age-specific death rates are regressed on the midpoint of the age interval and on the interaction of mid-point of the age interval with periods, separately for race/ethnicity and cause of death but using 2019 all-cause mortality as the reference category for both models. The slopes presented in this table are obtained as the sum of the baseline slope and the coefficient on the interaction between period and age, as such, they can be compared across models. The reported p-values are for two-sided significance tests on the interaction terms alone and as such measure the significance of deviations of period-specific slopes from the respective baseline. The open-ended age interval (85+) is excluded from the Gompertz model

**Table 3:** Ratios of race/ethnicity- and age-specific death rates during selected mortality periods

	All-cause						COVID-19				
	Baseline	Initial	Winter	Delta	Omicron	Endemic	Initial	Winter	Delta	Omicron	Endemic
	(2019)	(3/20–8/20)	(11/20–2/21)	(8/21–10/21)	(12/21–2/22)	(3/22–12/22)	(3/20–8/20)	(11/20–2/21)	(8/21–10/21)	(12/21–2/22)	(3/22–12/22)
**Black:White**											
35–39	1.46	1.60*	1.62*	1.68*	1.55*	1.58*	7.54*	3.11*	2.29*	1.78*	1.80*
40–44	1.52	1.75*	1.65*	1.67*	1.67*	1.58	9.68*	2.81*	2.02*	1.68*	1.74
45–49	1.48	1.79*	1.63*	1.63*	1.57*	1.53*	7.24*	2.32*	1.84*	1.50	1.65
50–54	1.46	1.74*	1.65*	1.55*	1.46	1.47	6.71*	2.46*	1.61*	1.33^	1.44
55–59	1.47	1.73*	1.65*	1.49	1.45	1.45	5.68*	2.29*	1.47	1.38^	1.19^
60–64	1.51	1.81*	1.67*	1.53	1.49	1.46^	5.57*	1.97*	1.56	1.36^	1.28^
65–69	1.50	1.85*	1.66*	1.53	1.49	1.45^	5.18*	1.93*	1.53	1.42^	1.24^
70–74	1.33	1.68*	1.48*	1.35	1.37*	1.33	4.50*	1.65*	1.38	1.37	1.13^
75–79	1.18	1.49*	1.28*	1.17	1.19	1.15^	3.81*	1.33*	1.25*	1.22	0.99^
80–84	1.06	1.32*	1.10*	1.06	1.08	1.00^	2.92*	1.09	1.20*	1.17*	0.82^
85+	0.85	1.05*	0.89*	0.85	0.88*	0.81^	1.94*	0.81^	0.98*	1.108	0.67^
**Hispanic: White**											
35–39	0.65	0.81*	0.83*	0.91*	0.80*	0.77*	8.57*	4.17*	1.74*	1.33*	0.798*
40–44	0.66	0.86*	0.92*	0.90*	0.82*	0.73*	10.00*	3.91*	1.63*	1.32*	0.868*
45–49	0.67	0.90*	0.99*	0.86*	0.78*	0.72*	7.92*	3.63*	1.39*	1.13*	0.73
50–54	0.67	0.94*	1.07*	0.83*	0.75*	0.69*	7.18*	3.60*	1.24*	1.05*	0.818*
55–59	0.69	0.97*	1.14*	0.81*	0.76*	0.69	6.42*	3.71*	1.22*	1.04*	0.88*
60–64	0.71	1.01*	1.18*	0.82*	0.79*	0.70	5.69*	3.33*	1.29*	1.12*	0.86*
65–69	0.76	1.08*	1.25*	0.86*	0.81*	0.74	5.14*	3.19*	1.31*	1.11*	0.96*
70–74	0.76	1.06*	1.16*	0.81*	0.84*	0.77	4.29*	2.48*	1.19*	1.18*	0.83*
75–79	0.74	1.00*	1.07*	0.80*	0.82*	0.73	3.31*	1.96*	1.16*	1.11*	0.71
80–84	0.71	0.94*	0.95*	0.77*	0.78*	0.71	2.55*	1.54*	1.01*	1.05*	0.75
85+	0.68	0.82*	0.78*	0.71*	0.75*	0.66^	1.63*	0.99*	0.96*	1.06*	0.66
**Asian: White**											
35–39	0.28	0.30	0.32	0.25	0.27	0.28	2.05*	1.37*	0.31	0.34	0.40
40–44	0.31	0.33	0.38*	0.30	0.30	0.32	1.86*	1.08*	0.36	0.33	0.54*
45–49	0.36	0.41*	0.41*	0.32^	0.34	0.35	1.80*	0.92*	0.27^	0.32	0.31
50–54	0.39	0.44*	0.48*	0.35^	0.37	0.39	1.97*	0.93*	0.30^	0.29^	0.45
55–59	0.39	0.46*	0.48*	0.35^	0.38	0.37	1.85*	1.00*	0.24^	0.36	0.39
60–64	0.42	0.51*	0.56*	0.38^	0.39^	0.41	1.99*	1.16*	0.31^	0.36^	0.41
65–69	0.45	0.56*	0.62*	0.42^	0.46	0.45	1.85*	1.15*	0.35^	0.39^	0.50
70–74	0.48	0.58*	0.63*	0.45^	0.48	0.50*	1.54*	1.00*	0.34^	0.49	0.48
75–79	0.53	0.60*	0.62*	0.49^	0.50	0.53	1.32*	0.84*	0.34^	0.48^	0.47^
80–84	0.56	0.64*	0.67*	0.54	0.58	0.56	1.13*	0.87*	0.35^	0.56	0.58
85+	0.58	0.65*	0.65*	0.55^	0.60*	0.57	0.97*	0.71*	0.36^	0.60	0.61*

*T* tests are one-sided and conducted at 95% level

* (^) indicates where age-specific death rate ratio is statistically greater (smaller) than all-cause death rate ratio at baseline (2019)
