# Supplementary material for: COVID-19 and All-Cause Mortality by Race, Ethnicity, and Age Across Five Periods of the Pandemic in the United States
Source: Popul Res Policy Rev. Author manuscript; Available in PMC 2023 Sep 29. (PMC10540502; doi:10.1007/s11113-023-09817-8)
Supplement: Supplementary-Appendix [file NIHMS1932798-supplement-Supplementary-Appendix.docx]

**Supplementary Appendix**

**Table S1: 95% confidence intervals of race/ethnicity and age-specific death rates ratios**

|  | **Baseline** | | **Initial** | | **Winter** | | **Delta** | | **Omicron** | | **Endemic** | |
| --- | --- | --- | --- | --- | --- | --- | --- | --- | --- | --- | --- | --- |
|  | (2019) | | (3/20-8/20) | | (11/20-2/21) | | (8/21-10/21) | | (12/21-2/22) | | (3/22-12/22) | |
|  | Ratio | 95% CI | Ratio | 95% CI | Ratio | 95% CI | Ratio | 95% CI | Ratio | 95% CI | Ratio | 95% CI |
| **All-Cause** |  |  |  |  |  |  |  |  |  |  |  |  |
| Black:White |  |  |  |  |  |  |  |  |  |  |  |  |
| 35-39 | 1.46 | (1.42, 1.49) | 1.60 | (1.55, 1.65) | 1.62 | (1.56, 1.68) | 1.68 | (1.61, 1.75) | 1.55 | (1.49, 1.62) | 1.58 | (1.54, 1.62) |
| 40-44 | 1.52 | (1.49, 1.56) | 1.75 | (1.71, 1.81) | 1.65 | (1.59, 1.71) | 1.67 | (1.61, 1.73) | 1.67 | (1.61, 1.73) | 1.58 | (1.54, 1.61) |
| 45-49 | 1.48 | (1.45, 1.51) | 1.79 | (1.75, 1.83) | 1.63 | (1.58, 1.67) | 1.63 | (1.58, 1.68) | 1.57 | (1.51, 1.62) | 1.53 | (1.5, 1.56) |
| 50-54 | 1.46 | (1.44, 1.49) | 1.74 | (1.71, 1.78) | 1.65 | (1.61, 1.69) | 1.55 | (1.52, 1.6) | 1.46 | (1.42, 1.5) | 1.47 | (1.45, 1.5) |
| 55-59 | 1.47 | (1.45, 1.49) | 1.73 | (1.7, 1.75) | 1.65 | (1.62, 1.68) | 1.49 | (1.46, 1.52) | 1.45 | (1.42, 1.48) | 1.45 | (1.43, 1.47) |
| 60-64 | 1.51 | (1.49, 1.53) | 1.81 | (1.79, 1.84) | 1.67 | (1.64, 1.7) | 1.53 | (1.5, 1.55) | 1.49 | (1.46, 1.52) | 1.46 | (1.44, 1.47) |
| 65-69 | 1.50 | (1.48, 1.51) | 1.85 | (1.83, 1.87) | 1.66 | (1.64, 1.69) | 1.53 | (1.5, 1.55) | 1.49 | (1.47, 1.52) | 1.45 | (1.44, 1.47) |
| 70-74 | 1.33 | (1.32, 1.34) | 1.68 | (1.66, 1.7) | 1.48 | (1.46, 1.5) | 1.35 | (1.33, 1.37) | 1.37 | (1.35, 1.39) | 1.33 | (1.32, 1.35) |
| 75-79 | 1.18 | (1.17, 1.2) | 1.49 | (1.47, 1.5) | 1.28 | (1.26, 1.29) | 1.17 | (1.15, 1.19) | 1.19 | (1.17, 1.21) | 1.15 | (1.14, 1.17) |
| 80-84 | 1.06 | (1.05, 1.07) | 1.32 | (1.3, 1.33) | 1.10 | (1.09, 1.12) | 1.06 | (1.05, 1.08) | 1.08 | (1.06, 1.09) | 1.00 | (0.99, 1.01) |
| 85+ | 0.85 | (0.85, 0.86) | 1.05 | (1.04, 1.05) | 0.89 | (0.88, 0.9) | 0.85 | (0.84, 0.86) | 0.88 | (0.87, 0.88) | 0.81 | (0.8, 0.81) |
| Hispanic:White |  |  |  |  |  |  |  |  |  |  |  |  |
| 35-39 | 0.65 | (0.63, 0.67) | 0.81 | (0.78, 0.83) | 0.83 | (0.8, 0.87) | 0.91 | (0.87, 0.95) | 0.80 | (0.77, 0.84) | 0.77 | (0.75, 0.79) |
| 40-44 | 0.66 | (0.64, 0.68) | 0.86 | (0.83, 0.89) | 0.92 | (0.89, 0.95) | 0.90 | (0.87, 0.93) | 0.82 | (0.78, 0.85) | 0.73 | (0.71, 0.75) |
| 45-49 | 0.67 | (0.66, 0.69) | 0.90 | (0.87, 0.92) | 0.99 | (0.96, 1.03) | 0.86 | (0.84, 0.89) | 0.78 | (0.75, 0.8) | 0.72 | (0.7, 0.73) |
| 50-54 | 0.67 | (0.66, 0.68) | 0.94 | (0.92, 0.96) | 1.07 | (1.04, 1.1) | 0.83 | (0.81, 0.86) | 0.75 | (0.73, 0.78) | 0.69 | (0.68, 0.71) |
| 55-59 | 0.69 | (0.68, 0.7) | 0.97 | (0.95, 0.99) | 1.14 | (1.12, 1.16) | 0.81 | (0.79, 0.83) | 0.76 | (0.74, 0.78) | 0.69 | (0.67, 0.7) |
| 60-64 | 0.71 | (0.7, 0.73) | 1.01 | (1, 1.03) | 1.18 | (1.16, 1.21) | 0.82 | (0.8, 0.84) | 0.79 | (0.77, 0.81) | 0.70 | (0.69, 0.71) |
| 65-69 | 0.76 | (0.74, 0.77) | 1.08 | (1.06, 1.1) | 1.25 | (1.23, 1.28) | 0.86 | (0.84, 0.88) | 0.81 | (0.79, 0.83) | 0.74 | (0.73, 0.75) |
| 70-74 | 0.76 | (0.75, 0.77) | 1.06 | (1.04, 1.08) | 1.16 | (1.14, 1.18) | 0.81 | (0.79, 0.83) | 0.84 | (0.82, 0.85) | 0.77 | (0.76, 0.78) |
| 75-79 | 0.74 | (0.73, 0.75) | 1.00 | (0.98, 1.01) | 1.07 | (1.05, 1.08) | 0.80 | (0.78, 0.82) | 0.82 | (0.8, 0.83) | 0.73 | (0.72, 0.74) |
| 80-84 | 0.71 | (0.7, 0.72) | 0.94 | (0.93, 0.95) | 0.95 | (0.94, 0.97) | 0.77 | (0.75, 0.78) | 0.78 | (0.76, 0.79) | 0.71 | (0.7, 0.72) |
| 85+ | 0.68 | (0.68, 0.69) | 0.82 | (0.81, 0.83) | 0.78 | (0.78, 0.79) | 0.71 | (0.7, 0.72) | 0.75 | (0.74, 0.76) | 0.66 | (0.66, 0.66) |
| Asian:White |  |  |  |  |  |  |  |  |  |  |  |  |
| 35-39 | 0.28 | (0.26, 0.3) | 0.30 | (0.28, 0.33) | 0.32 | (0.29, 0.35) | 0.25 | (0.22, 0.28) | 0.27 | (0.24, 0.31) | 0.28 | (0.26, 0.3) |
| 40-44 | 0.31 | (0.29, 0.33) | 0.33 | (0.31, 0.36) | 0.38 | (0.35, 0.41) | 0.30 | (0.27, 0.33) | 0.30 | (0.27, 0.33) | 0.32 | (0.3, 0.33) |
| 45-49 | 0.36 | (0.34, 0.38) | 0.41 | (0.38, 0.43) | 0.41 | (0.38, 0.44) | 0.32 | (0.29, 0.34) | 0.34 | (0.32, 0.37) | 0.35 | (0.33, 0.37) |
| 50-54 | 0.39 | (0.37, 0.41) | 0.44 | (0.41, 0.46) | 0.48 | (0.45, 0.51) | 0.35 | (0.32, 0.37) | 0.37 | (0.34, 0.4) | 0.39 | (0.37, 0.4) |
| 55-59 | 0.39 | (0.38, 0.41) | 0.46 | (0.44, 0.48) | 0.48 | (0.46, 0.51) | 0.35 | (0.32, 0.37) | 0.38 | (0.36, 0.4) | 0.37 | (0.36, 0.39) |
| 60-64 | 0.42 | (0.41, 0.44) | 0.51 | (0.5, 0.53) | 0.56 | (0.54, 0.58) | 0.38 | (0.36, 0.4) | 0.39 | (0.37, 0.41) | 0.41 | (0.4, 0.42) |
| 65-69 | 0.45 | (0.44, 0.47) | 0.56 | (0.55, 0.58) | 0.62 | (0.6, 0.64) | 0.42 | (0.4, 0.44) | 0.46 | (0.44, 0.48) | 0.45 | (0.44, 0.46) |
| 70-74 | 0.48 | (0.46, 0.49) | 0.58 | (0.56, 0.59) | 0.63 | (0.61, 0.65) | 0.45 | (0.43, 0.46) | 0.48 | (0.47, 0.5) | 0.50 | (0.49, 0.51) |
| 75-79 | 0.53 | (0.52, 0.54) | 0.60 | (0.59, 0.62) | 0.62 | (0.61, 0.64) | 0.49 | (0.47, 0.51) | 0.50 | (0.49, 0.52) | 0.53 | (0.52, 0.54) |
| 80-84 | 0.56 | (0.55, 0.57) | 0.64 | (0.62, 0.65) | 0.67 | (0.65, 0.69) | 0.54 | (0.52, 0.56) | 0.58 | (0.56, 0.59) | 0.56 | (0.55, 0.57) |
| 85+ | 0.58 | (0.57, 0.58) | 0.65 | (0.65, 0.66) | 0.65 | (0.64, 0.65) | 0.55 | (0.54, 0.56) | 0.60 | (0.59, 0.61) | 0.57 | (0.57, 0.58) |

**Table S1: 95% confidence intervals of race/ethnicity and age-specific death rates ratios… continued**

|  | **Baseline** | | **Initial** | | **Winter** | | **Delta** | | **Omicron** | | **Endemic** | |
| --- | --- | --- | --- | --- | --- | --- | --- | --- | --- | --- | --- | --- |
|  | (2019) | | (3/20-8/20) | | (11/20-2/21) | | (8/21-10/21) | | (12/21-2/22) | | (3/22-12/22) | |
|  | Ratio | 95% CI | Ratio | 95% CI | Ratio | 95% CI | Ratio | 95% CI | Ratio | 95% CI | Ratio | 95% CI |
| **COVID-19** |  |  |  |  |  |  |  |  |  |  |  |  |
| Black:White |  |  |  |  |  |  |  |  |  |  |  |  |
| 35-39 | - | | 7.54 | (6.39, 8.88) | 3.11 | (2.72, 3.62) | 2.29 | (2.09, 2.48) | 1.77 | (1.57, 1.98) | 1.95 | (1.52, 2.43) |
| 40-44 | - | | 9.68 | (8.5, 11.1) | 2.81 | (2.53, 3.13) | 2.02 | (1.88, 2.17) | 1.67 | (1.51, 1.84) | 1.79 | (1.48, 2.16) |
| 45-49 | - | | 7.24 | (6.59, 7.95) | 2.32 | (2.13, 2.51) | 1.84 | (1.74, 1.95) | 1.49 | (1.38, 1.61) | 1.50 | (1.24, 1.76) |
| 50-54 | - | | 6.71 | (6.3, 7.17) | 2.46 | (2.33, 2.61) | 1.61 | (1.52, 1.69) | 1.32 | (1.24, 1.41) | 1.42 | (1.24, 1.61) |
| 55-59 | - | | 5.68 | (5.39, 6.01) | 2.29 | (2.18, 2.39) | 1.47 | (1.4, 1.53) | 1.38 | (1.31, 1.45) | 1.10 | (0.98, 1.24) |
| 60-64 | - | | 5.57 | (5.35, 5.8) | 1.97 | (1.89, 2.05) | 1.56 | (1.5, 1.63) | 1.35 | (1.3, 1.41) | 1.25 | (1.15, 1.36) |
| 65-69 | - | | 5.18 | (4.99, 5.37) | 1.93 | (1.87, 2) | 1.53 | (1.47, 1.59) | 1.41 | (1.35, 1.47) | 1.15 | (1.06, 1.24) |
| 70-74 | - | | 4.50 | (4.36, 4.64) | 1.65 | (1.6, 1.7) | 1.38 | (1.32, 1.44) | 1.36 | (1.3, 1.41) | 1.09 | (1.02, 1.17) |
| 75-79 | - | | 3.81 | (3.69, 3.91) | 1.33 | (1.29, 1.37) | 1.25 | (1.19, 1.31) | 1.21 | (1.16, 1.26) | 0.96 | (0.89, 1.03) |
| 80-84 | - | | 2.92 | (2.83, 3) | 1.09 | (1.06, 1.13) | 1.20 | (1.14, 1.26) | 1.17 | (1.12, 1.22) | 0.83 | (0.77, 0.89) |
| 85+ | - | | 1.94 | (1.89, 1.98) | 0.81 | (0.79, 0.83) | 0.98 | (0.93, 1.02) | 1.10 | (1.07, 1.14) | 0.67 | (0.64, 0.7) |
| Hispanic:White |  |  |  |  |  |  |  |  |  |  |  |  |
| 35-39 | - | | 8.57 | (7.37, 9.94) | 4.17 | (3.74, 4.68) | 1.74 | (1.62, 1.88) | 1.32 | (1.19, 1.47) | 0.90 | (0.68, 1.14) |
| 40-44 | - | | 10.00 | (8.81, 11.43) | 3.91 | (3.59, 4.28) | 1.63 | (1.52, 1.75) | 1.33 | (1.22, 1.45) | 0.89 | (0.71, 1.09) |
| 45-49 | - | | 7.92 | (7.29, 8.62) | 3.63 | (3.42, 3.89) | 1.39 | (1.32, 1.47) | 1.13 | (1.05, 1.21) | 0.77 | (0.64, 0.92) |
| 50-54 | - | | 7.18 | (6.74, 7.68) | 3.60 | (3.42, 3.78) | 1.24 | (1.17, 1.3) | 1.05 | (0.98, 1.11) | 0.90 | (0.78, 1.02) |
| 55-59 | - | | 6.42 | (6.11, 6.72) | 3.71 | (3.58, 3.84) | 1.22 | (1.16, 1.27) | 1.04 | (0.98, 1.09) | 0.95 | (0.85, 1.06) |
| 60-64 | - | | 5.69 | (5.47, 5.92) | 3.33 | (3.23, 3.44) | 1.29 | (1.23, 1.34) | 1.12 | (1.07, 1.17) | 0.93 | (0.84, 1.03) |
| 65-69 | - | | 5.14 | (4.97, 5.34) | 3.19 | (3.1, 3.27) | 1.31 | (1.25, 1.37) | 1.11 | (1.06, 1.16) | 0.98 | (0.89, 1.06) |
| 70-74 | - | | 4.29 | (4.15, 4.45) | 2.48 | (2.42, 2.54) | 1.19 | (1.13, 1.25) | 1.17 | (1.12, 1.22) | 0.91 | (0.84, 0.98) |
| 75-79 | - | | 3.31 | (3.2, 3.42) | 1.96 | (1.91, 2.01) | 1.16 | (1.1, 1.21) | 1.11 | (1.06, 1.16) | 0.77 | (0.7, 0.82) |
| 80-84 | - | | 2.55 | (2.47, 2.64) | 1.54 | (1.5, 1.58) | 1.01 | (0.96, 1.07) | 1.05 | (1.01, 1.1) | 0.78 | (0.72, 0.84) |
| 85+ | - | | 1.63 | (1.59, 1.66) | 0.99 | (0.97, 1.02) | 0.96 | (0.92, 1.01) | 1.06 | (1.02, 1.09) | 0.66 | (0.64, 0.69) |
| Asian:White |  |  |  |  |  |  |  |  |  |  |  |  |
| 35-39 | - | | 2.05 | (1.52, 2.61) | 1.37 | (1.08, 1.67) | 0.31 | (0.24, 0.39) | 0.33 | (0.23, 0.42) | 0.38 | (0.19, 0.62) |
| 40-44 | - | | 1.86 | (1.46, 2.34) | 1.08 | (0.89, 1.29) | 0.36 | (0.29, 0.43) | 0.33 | (0.26, 0.41) | 0.60 | (0.36, 0.83) |
| 45-49 | - | | 1.80 | (1.49, 2.15) | 0.92 | (0.79, 1.07) | 0.27 | (0.23, 0.32) | 0.32 | (0.26, 0.38) | 0.32 | (0.18, 0.45) |
| 50-54 | - | | 1.97 | (1.71, 2.21) | 0.93 | (0.82, 1.04) | 0.30 | (0.25, 0.34) | 0.29 | (0.24, 0.34) | 0.43 | (0.31, 0.57) |
| 55-59 | - | | 1.85 | (1.66, 2.04) | 1.00 | (0.91, 1.09) | 0.24 | (0.2, 0.27) | 0.37 | (0.32, 0.41) | 0.40 | (0.3, 0.5) |
| 60-64 | - | | 1.99 | (1.84, 2.16) | 1.16 | (1.08, 1.24) | 0.31 | (0.27, 0.35) | 0.36 | (0.32, 0.41) | 0.42 | (0.33, 0.51) |
| 65-69 | - | | 1.85 | (1.72, 1.98) | 1.15 | (1.09, 1.21) | 0.35 | (0.31, 0.39) | 0.38 | (0.34, 0.42) | 0.51 | (0.43, 0.58) |
| 70-74 | - | | 1.54 | (1.44, 1.64) | 1.00 | (0.95, 1.05) | 0.34 | (0.3, 0.38) | 0.49 | (0.45, 0.53) | 0.51 | (0.44, 0.58) |
| 75-79 | - | | 1.32 | (1.24, 1.41) | 0.84 | (0.8, 0.88) | 0.34 | (0.3, 0.38) | 0.48 | (0.44, 0.52) | 0.47 | (0.41, 0.53) |
| 80-84 | - | | 1.13 | (1.07, 1.21) | 0.87 | (0.83, 0.91) | 0.35 | (0.31, 0.4) | 0.56 | (0.52, 0.6) | 0.66 | (0.6, 0.73) |
| 85+ | - | | 0.97 | (0.93, 1) | 0.71 | (0.69, 0.73) | 0.36 | (0.33, 0.4) | 0.60 | (0.57, 0.63) | 0.61 | (0.58, 0.64) |

Note: For details on the construction of the confidence intervals, please see process described in the Data & Methods section.
